# Supplementary material for: Transcriptomic Response of the Diazotrophic Bacteria Gluconacetobacter diazotrophicus Strain PAL5 to Iron Limitation and Characterization of the fur Regulatory Network
Source: Int J Mol Sci. 2022 Aug 1;23(15):8533. doi: 10.3390/ijms23158533 (PMC9368920; doi:10.3390/ijms23158533)
Supplement: Supplementary file 1 [file ijms-23-08533-s001.zip › Figure S1.pdf]

0 — 1

	0 μM $FeCl_3$ R1	0 μM $FeCl_3$ R2	0 μM $FeCl_3$ R3	37 μM $FeCl_3$ R1	37 μM $FeCl_3$ R2	37 μM $FeCl_3$ R3
0 μM $FeCl_3$ R1		0.996643	0.993483	0.98085	0.981307	0.969821
0 μM $FeCl_3$ R2			0.99648	0.981497	0.983136	0.970178
0 μM $FeCl_3$ R3				0.975026	0.976645	0.964262
37 μM $FeCl_3$ R1					0.997879	0.995015
37 μM $FeCl_3$ R2						0.995844
37 μM $FeCl_3$ R3						

Figure S1. Correlation matrix of unique gene reads between biological replicates according to the identification of differentially expressed genes through transcriptomic analysis.
