# Supplementary material for: Transcriptomic Response of the Diazotrophic Bacteria Gluconacetobacter diazotrophicus Strain PAL5 to Iron Limitation and Characterization of the fur Regulatory Network
Source: Int J Mol Sci. 2022 Aug 1;23(15):8533. doi: 10.3390/ijms23158533 (PMC9368920; doi:10.3390/ijms23158533)
Supplement: Supplementary file 1 [file ijms-23-08533-s001.zip › Table S1.pdf]

Table S1. Data from the RNAseq analysis for *G. diazotrophicus* strain PAL5 grown in the presence and the absence of iron.

Samples	Number of reads for each biological replicate	Number of reads after removal of rRNAs	Number of reads mapped exclusively in the genome of the strain PAL5
0 μM $FeCl_3$ R1	3,519,415	902,167	461,910
0 μM $FeCl_3$ R2	4,929,282	1,277,238	613,074
0 μM $FeCl_3$ R3	6,735,220	1,790,305	823,540
**Total**	**15,183,917**	**3,969,710**	**1,898,524**
37 μM $FeCl_3$R1	5,109,801	1,873,467	805,591
37 μM $FeCl_3$R2	3,761,570	822,848	378,510
37 μM $FeCl_3$R3	4,722,612	2,422,989	1,090,345
**Total**	**13,593,983**	**5,119,304**	**2,274,446**

The reads were mapped to the genome of *G. diazotrophicus* strain PAL5 with the CLC Genomics Workbench v.7.5.1 software, with a minimum length of 90% and similarity of 80. The numbers R1, R2 and R3 refer to the replicates of the biological samples of 0 μM and 37 μM $FeCl_3$.
