# Supplementary material for: Transcriptomic Response of the Diazotrophic Bacteria Gluconacetobacter diazotrophicus Strain PAL5 to Iron Limitation and Characterization of the fur Regulatory Network
Source: Int J Mol Sci. 2022 Aug 1;23(15):8533. doi: 10.3390/ijms23158533 (PMC9368920; doi:10.3390/ijms23158533)
Supplement: Supplementary file 1 [file ijms-23-08533-s001.zip › Table S4.pdf]

Table S4. Primers used for validation of RNA-seq transcriptome by RT-qPCR.

Gene	Forward sequence (5'-3')<br>Reverse sequence (5'-3')	Amplicon length (bp)	Primer concentration (pmol)	Anneling temperature (°C)
pstC	GATTCTGGTTCTGATGGGCG<br>ACACCGTGCTCCACACAAAC	98	500	60
fxdB	CCGAGGACGAGGAAGACATG<br>GGTCATCACGATCTGGCAGC	80	500	60
groS	CGAACAGGTCGGATTCCTT<br>GTCCTGTTCGGGAAATGGT	82	500	60
wrbA	GCATCTGGCCCTGGAAATTATA<br>CCTGTTCTCCGTCATCACCAA	79	300	60
GDI_1972	CGATCTGGCTGGTCGAATGA<br>TCCACAGTATTTCCGGGCAC	87	300	60
GDI_0466	CGACCCCGAGATCATCATC<br>CTTGACCACGGATTTGAACG	94	300	60
