# Supplementary material for: Transcriptomic Response of the Diazotrophic Bacteria Gluconacetobacter diazotrophicus Strain PAL5 to Iron Limitation and Characterization of the fur Regulatory Network
Source: Int J Mol Sci. 2022 Aug 1;23(15):8533. doi: 10.3390/ijms23158533 (PMC9368920; doi:10.3390/ijms23158533)
Supplement: Supplementary file 1 [file ijms-23-08533-s001.zip › Table S5.pdf]

Table S5. Bacterial strains, plasmids and primers used in this study

Strains, plasmids and primers	Description and/or sequence	Source or reference
Strains		
G. diazotrophicus PAL5^T	Wild-type (BR11281)	Embrapa Agrobiology Culture Collection
E. coli DH10B	F– *mcr*A Δ(*mrr-hsd*RMS-*mcr*BC) Φ80*lac*ZΔM15 Δ*lac*X74 *rec*A1 *end*A1 *ara*D139 Δ(*ara leu*) 7697 *gal*U *gal*K *rps*L *nup*G λ–	Invitrogen™
E. coli H1780	araD139ΔaargF-lacU169rpsL150 relA1 flbB5301deoC1 ptsF25 *rbs*R *fiu::lacZ* fusion lacking Fur	Hantke, 1987
E. coli H1780 (pGGd*fur*)	*E. coli* H1780 carrying Gd*fur*	This study
E. coli H1780 (pGGd*furTn5*)	*E. coli* H1780 carrying Gd*furTn5*	This study
**Plasmids**		
pGEM®-T easy	High copy-number cloning vector; Amp^R	Promega
pGd*fur*	pGEM®-T easy containing Gd*fur*; Amp^R	This study
pGd*fur*::Tn*5*_A	pGEM®-T easy containing Gd*fur*::Tn*5*; Kan^R	This study
pGdfur::Tn*5*_B	pGEM®-T easy containing Gd*fur*::Tn*5*; Kan^R	This study
**Primers**		
Gdfur-F	5′ – AGCATATGGTAGCGGCAGGCGAGGC - 3′	This study
Gdfur-R	5′ – CGAAGCTTGCCGCCGTGATTCATCCTTG - 3′	This study
